# Phthisis and Compression of the Lung

**Published:** 1898-07-16

**Authors:** 


### PHTHISIS AND COMPRESSION OF THE LUNG.

With the object of treating tuberculosis of the lung on the principle of securing physiological rest for the diseased organ, Dr. Murphy, of Chicago, suggests a novel proceeding. As is well known, many believe that the pleurisy which so often complicates tuberculosis of the lungs is a sort of protective mechanism, and that the compression of the lung which is produced by large effusion into the pleura has an influence in retarding or holding in check the major disease. In fact, the idea has grown up, although it is held by some to be a mere prejudice, that tuberculous pleurisies should not be interfered with. Dr. Murphy, however, is so impressed with the importance of giving rest to a tuberculous organ that he proposes to obtain by surgical means that compression of the lung which he looks upon as so important an agent in stopping the progress of tuberculous disease in its tissues. He proposes, in fact, to fill up the pleura with sterile nitrogen, which, being very slowly absorbed, may be allowed to remain for months without injury to the patient—in fact, "until the disease is cured." There is but little doubt that Dr. Murphy is right when he says that "the prominence of tuberculosis of the lungs is due to the opportunities for infection, and not to lowered resistance," and, perhaps, this would be still more true if he spoke of opportunities for re-infection. The suggestion, of course, is that when the lung is collapsed these opportunities are put a stop to. "Rest favours resistance, and by the injection of nitrogen the diseased lung will be allowed to rest. Any functionary useless organ is the seat of connective tissue overgrowth. Thus, as the lung is a functionary organ at rest, these tissues rapidly form and enclose the destructive germs in a prison." It is said that Dr. Murphy has over two hundred consumptive patients who are willing to undergo the operation. What, indeed, is there that consumptives will not undergo if they can but receive a promise of a cure?

